# Current animal models of bladder cancer: Awareness of translatability (Review)

**DOI:** 10.3892/etm.2014.1837

**Published:** 2014-07-11

**Authors:** JIE DING, DING XU, CHUNWU PAN, MIN YE, JIAN KANG, QIANG BAI, JUN QI

**Affiliations:** Department of Urology, Xin Hua Hospital Affiliated to Shanghai Jiao Tong University School of Medicine, Yangpu, Shanghai 200092, P.R. China

**Keywords:** animal model, bladder cancer, carcinogen-induced, genetically engineered mouse, translatability

## Abstract

Experimental animal models are crucial in the study of biological behavior and pathological development of cancer, and evaluation of the efficacy of novel therapeutic or preventive agents. A variety of animal models that recapitulate human urothelial cell carcinoma have thus far been established and described, while models generated by novel techniques are emerging. At present a number of reviews on animal models of bladder cancer comprise the introduction of one type of method, as opposed to commenting on and comparing all classifications, with the merits of a certain method being explicit but the shortcomings not fully clarified. Thus the aim of the present study was to provide a summary of the currently available animal models of bladder cancer including transplantable (which could be divided into xenogeneic or syngeneic, heterotopic or orthotopic), carcinogen-induced and genetically engineered models in order to introduce their materials and methods and compare their merits as well as focus on the weaknesses, difficulties in operation, associated problems and translational potential of the respective models. Findings of these models would provide information for authors and clinicians to select an appropriate model or to judge relevant preclinical study findings. Pertinent detection methods are therefore briefly introduced and compared.

## 1. Introduction

Bladder cancer is one of the most common types of cancer globally. An estimated 72,570 new cases and 15,210 mortalities from bladder cancer occurred in 2013 worldwide (1). The majority of types of bladder cancer arise from the urothelium, the well-differentiated transitional epithelium that lines the urinary bladder, also known as urothelial cell carcinoma (UCC). Bladder cancer is the most common malignancy of the urinary tract and the second most common malignancy of the urogenital tract following prostate cancer in the United States (2). Risk factors that have been clearly associated with the formation of urothelial tumors include tobacco smoking, occupational exposure to aromatic amines, consumption of arsenic-laced water and the therapeutic use of alkylating agents. Of all UCCs, 70% are a low-grade, papillary, non-invasive entity that tends to recur in more than half of patients following local resection. With regard to the progression of low-grade tumors into invasive forms, certain studies suggested that the progression rate may be as high as 10–20% (3,4). However, controversy as to whether this is true progression or *de novo* development remains. The remaining 20–30% of UCC are a high-grade, non-papillary, muscle-invasive form that are responsible for the majority of morbidity and mortality. These invasive tumors can penetrate deeply through the muscle wall of the bladder, demonstrating a high propensity for distant metastasis and resistance to currently available treatments (5). These invasive and non-invasive forms of UCC, are not only clinically distinguishable, but also reflected in two divergent molecular pathways (6,7), while the majority of epithelial tumors are believed to progress along a single pathway.

Although certain progress has been achieved in exploring the molecular basis that underlies UCC, in general it is not thoroughly understood and bladder cancer remains a major public health issue. The effects of conventional radiotherapy and chemotherapy on advanced bladder cancer remain unsatisfactory, therefore there remains a demand for the emergence of novel agents either for survival improvement or for chemoprevention. Among all types of models of bladder cancer which contribute to our understanding of the cancer itself or novel therapies, animal models have an important role that cannot be replaced by other models. They constitute the essential link between cell-based experiments and clinical trials, allowing the investigation of aspects that cannot be studied in traditional two-dimensional cell culture or clinical conditions, including the biological behavior and pathological characteristics of cancer in living organisms, the genotypic and phenotypic heterogeneity among a cluster of cancer cells, or the efficacy, toxicity and pharmacokinetics of a novel medicine. The invention of artificial three-dimensional extracellular matrices partly solved certain issues in terms of histological structures, cell differentiation and adhesion (8–13). However, cell cultures usually harbor only one type of cell, while real tumors constitute tumor, normal functional, stromal and immune cells, as well as other cell types that may support, promote or inhibit tumor growth. The microenvironment in cell culture regarding chemical components, biological properties and cell-to-cell interaction remains far from the *in vivo* condition. Increased effort has also been directed to the culture of normal tissues and organs, rather than to tumors and types of cancer.

For bladder cancer particularly, in the past century, much effort has been devoted to the development of an appropriate animal model. Certain criteria, however, are required for an ideal animal model, including that the tumor be of urothelial origin; mimic human bladder cancer pathogenesis; be orthotopic, meaning growing inside the urinary bladder instead of elsewhere; have several distinguishable pathological stages, similar to human bladder cancer; have similar phenotypic and genotypic alterations to those found in human bladder cancer, preserving heterogeneity at the same time; the animal host should be immunocompetent and easily manipulated from any aspect; and predominantly, the animal model should be feasible, in other words easy to set up, fast to develop, inexpensive to maintain, stable, predictable and reproducible. Unfortunately, the existing models barely adhere to these standards. Each animal model has its own advantages and disadvantages, and is only suitable for certain fields of study. In this review a critical look is taken at representatives of each type of animal model.

## 2. Selection of animals

Large mammalian animals including dogs or monkeys are biologically closer to humans, however, these induce financial burden and ethical issues. At present the most commonly used animal for bladder cancer models are rodents, including rats and mice. Rodents have a lower urinary tract comparable to humans, although neoplasms in the bladder are morphologically alike (14). Despite the large difference in size, 80% of mouse genes may be matched to human genes, and the ratio for the rat is 90% (15). Additionally, these small animals are fast to grow and reproduce, inexpensive to acquire and maintain, and convenient for manipulation. Their robust vitality allows them to endure operations and resist mild infections. Although imperfect in their translatability into clinical knowledge, the rodents remain a critical tool in bladder cancer research.

## 3. Transplantable models

### Xenogeneic models

Xenograft models are established by transplanting human urothelial cancer cell lines or primary cancer tissue fragments into immunodeficient mice (16). The receiver has to be the nude mouse model, which was identified in 1962 by Dr N.R. Grist (17). Lacking a thymus, nude mice cannot generate mature T lymphocytes and are unable to mount the majority of types of immune response, otherwise the strong rejection response in immunocompetent mice would lead to either the mortality of the mice or the death of the cancer cells.

A number of cell lines, derived from human primary UCC tissue by immortalization or spontaneous proliferation *in vitro*, representing tumors of different grades and stages, have been developed and had their histology and tumorigenicity variably characterized. The cell lines have been shown to retain the characteristics of the originating tumors (18), particularly exemplified in xenograft models (19,20), and human *in vitro* organotypic systems (21).

However, cell lines may differ from their origins in morphology, phenotype or growth patterns (22–25). To a certain degree, they can be considered ‘abnormal’ simply by having undergone the immortalization process. High rates of passaging are associated with increased spontaneous mutations, senescence and selection processes (26,27). The heterogeneity and biodiversity of the primary tumor are also gradually lost during the passages. Tumors formed in the xenograft model are unable to comprise enough various cell types as in the natural human UCC.

Apart from the phenotypic and genotypic drifts due to the selection pressures imposed during extensive cell culture, cross-contaminated sub-lines, signifying one cell line that is unintentionally replaced by another similar cell line, occur occasionally (28). The difference in aspects including enzyme production, drug resistance and radio sensitivity could lead to confounding results. Morphological changes and a shift in ploidy are also exhibited in serial passages of subcutaneous UCC xenografts, which results in removing certain animals out of a trial and transplanting the tumor into new animals (29).

Several decades have passed since the first cancer cell line was cultivated, yet certain basic cell activities have not been thoroughly elucidated, and information on gene expression and alterations of specific cell lines remain insufficient. Despite these potential drawbacks, the application of human UCC cell lines rather than animal cancer cells appears pertinent and convincing, thus xenograft models are employed in a number of clinical studies on the efficacy of therapeutic agents, including cytotoxic drugs, immunosuppressants, radiotherapy and monoclonal antibodies (30–33). Within one to two weeks of implantation, urothelial neoplasm grows to a size sufficient for treatment evaluation, rendering it time- and cost-effective when compared with carcinogen-induced or transgenic mouse models. The inhibited immune system allows nude mice to receive cell or tissue grafts, but also leads to the uncontrollable growth of the cancer cells, invasion of the surrounding tissue, fast angiogenesis and rapid distant metastasis. In certain studies, xenograft models are used to simulate metastatic bladder cancer, studying neutralizing monoclonal antibody targeted at murine vascular endothelial growth factor receptor (30,34). Due to the immunodeficiency of nude mice, xenograft models are not suitable for studies on interactions between the host immune system and the tumor, therefore experiments associated with Bacillus Calmette-Guerin (BCG) cannot be carried out on such models. By contrast, transplantable syngeneic animal models are more appropriate to approach these issues. Nevertheless, these two models are not useful for the study of the mechanisms associated with carcinogenesis or the efficacy of agents in preventing carcinogenesis.

### Syngeneic models

In contrast to xenogeneic models, syngeneic models are established by inoculating rodent bladder cancer to syngeneic, immunocompetent animals. Syngeneic models are employed when the study focuses on the immune response or gene therapy. The commonly used rodent bladder cancer cell lines include AY-27, MBT-2 and MB49. AY-27 and MBT-2 were initially induced by feeding C3H/He mouse and Fischer 344 rat strains with N-[4-(5-nitro-2-furyl)-2-thiazolyl] formamide (FANFT) (35,36), while MB49 was induced by administering 7,12-dimethylbenzanthracene to the C57BL/6 mouse strain (37). However, one type of rodent cancer cell line may not be compatible to the rodents of other strains from which the cell line was originally derived (38,39).

Transplantable syngeneic models and xenograft models are both time- and cost-effective, and reproducible, but exhibit phenotypic and genotypic drifts in continuous cell or animal passages. The application of the immunocompetent host in syngeneic models allows the study of intravesical BCG treatment or gene therapy (19,40–45).

The immunoregulator BCG is widely recognized as a potent agent in preventing tumor recurrence, more effectively than any other chemotherapeutic agent. However, ~20% of patients discontinue BCG due to local and systemic toxicity and >30% of patients show evidence of recurrence (46), which has led to increased interest in exploring BCG-associated mechanisms (47). In this case, syngeneic animal bladder cancer models are essentially required as they provide a practical platform for seeking the reason underlying the lack of response of BCG observed in certain patients and testing the efficacy of combining new adjuvant agents with BCG (48–50).

Certain individuals argue that the tumors are of rodent and not human origin, and therefore inherent characteristics including tumor growth, latency, growth rate, invasion and metastasis may be different from their human counterparts, and therefore that the translational potential of syngeneic models is even lower than xenogeneic models, and is one step further from clinical applicability.

### Heterotopic models

Based on whether the inoculation site is in the target organ or not, xenogeneic and syngeneic models could be further divided into heterotopic and orthotopic models. Heterotopic indicates that the graft is not transplanted in the original site, but usually subcutaneously in the flank or hind leg of the animal. If the target organ is not convenient for inoculation such as the bladder, kidney or bowels, the subcutaneous model should be considered since it is technically simple and can be carried out by an operator with minimum training. Furthermore, the tumor can be easily and non-invasively detected, and the tumor evolution can be conveniently assessed by palpation of the skin and measured with a caliper. For these reasons, subcutaneous bladder tumor models have been widely used in assessing the efficacy of novel therapeutic agents (51).

However, the alteration of the tumor microenvironment due to the inoculation site may significantly affect the biological behavior of tumor growth and metastasis, genetic expression or the efficacy of anti-proliferative agents (52–54). Drugs that have great anti-proliferative or apoptosis-inducing potential on heterotopic animal models may not be effective on orthotopic models. In a well-known study (54), the highly metastatic KM12L4 human colon cancer cell line was implanted into different anatomical locations including the subcutis, cecum and spleen (leading to experimental liver metastasis) of nude mice, and doxorubicin was injected intravenously at 10 mg/kg for evaluation. Tumors grown within the subcutis showed an 80% inhibition of growth following two intravenous injections of doxorubicin, compared with only 40% inhibition of the intracecal tumors and <10% inhibition of lesions in the liver (54). Apart from their limited translatability, in the study of bladder cancer, the modalities of intravesical treatment cannot be directly applied to subcutaneous tumors, requiring the establishment of orthotopic models.

### Orthotopic models

Compared with subcutaneous models, orthotopic tumors mimic human bladder cancer behavior more closely, since the microenvironment is closer to the natural condition. Therefore experimental results generated by the orthotopic model are expected to have higher relevance (55). However, the accurate non-invasive assessment of established tumors in the orthotopic model also requires further detection devices.

Different methods of orthotopic model establishment have been reported, mostly by intravesical instillation of the tumor/cancer cell suspension following preconditioning of the urothelium. Tumor cells instilled into normal bladders did not result in tumor establishment. To damage the integrity of the urothelium, chemical denudation with HCl and subsequent neutralization with KOH or extensive washing with saline results in increased take rates (56,57). Other agents, e.g., N-methyl-N-nitrosurea (MNU) or silver nitrate can also be used for denudation but are not as effective as acid washing (36). Mechanical damage, including electrical cauterization or epithelial abrasion can also facilitate tumor cell adhesion (19,40,58). Prolonged dwell time of tumor cells can also improve take rates (19). Intramural inoculation via laparotomy does not require preconditioning of the urothelium or prolonged dwell time, and results in high take rates at the same time (59). However, the biological behavior of inoculated tumors may be altered compared with those inoculated by instillation (60). This does not signify that tumors inoculated through intravesical instillation of cell suspension completely mimic human bladder cancer. Certain tumors tend to be invasive from the beginning, without the Ta stages, possibly associated with preconditional urothelium damage such as extensive abrasion rather than with the aggressiveness of the cell line (61). Caution should be paid during bladder preconditioning to prevent the rapid invasion of tumor cells into the underlying layers of the bladder.

Following a period of one to two weeks, tumor growth is usually detectable (62,63). Signs indicating successful inoculation of the tumor include gross hematuria, significant weight loss and suprapubic mass. Palpation is easy and cost-free, but does not provide reliable parameters for evaluating the development of the tumor, and only large tumors (>200 mg) may be detected by palpation (49).

Ultrathin cystoscopy (diameter 0.75 mm), developed by Asanuma *et al*, is a reliable non-invasive method, permitting inspection of the urethra and whole bladder surface, obtaining detailed appearance and accurate locations of early lesions (64) with a sensitivity and specificity >90% (65). If enhancing agents such as aminolevulinic acid are used the result should be further improved (66). Cystoscopy does not distinguish non-muscle-invasive bladder cancer from muscle-invasive disease and does not provide quantitative analysis.

Quantitative analyses are necessary for the confirmation of inoculation and serial assessment of tumor evolution. Intravesical ultrasonography is reported as a reliable and appropriate non-invasive method for evaluating tumor stage and size in an orthotopic model with the positive predictive ratio regarding tumor staging reaching 85% (67). However, information on tumor location and appearance cannot be obtained. Resolution is limited and the detection procedure is skillfully demanding. Magnetic resonance imaging has been reported as an effective tool in terms of quantification (57,68,69). Tumors can be clearly delineated from normal tissue and their size can be accurately measured. In spite of all the merits, it offers poor diagnosis of small early lesions (<1 mm in diameter) due to its spatial resolution and slice thickness, and the detection process is relatively time-consuming.

All of the imaging tests require anaesthesia and urethral catheterization. A certain percentage of the animals (≤20%), may die prior to the end of the study due to procedural mishaps (70). Thus more animals are required from the beginning of the studies in case of accidental mortality during these tests. A combination of the detection methods introduced above can provide important information for records of tumor growth without sacrificing the animals, although pathological analysis of the bladder remains the gold standard.

## 4. Carcinogen-induced models

Urinary bladder carcinogenesis has been studied extensively ever since the report by the surgeon Dr Rehn suggesting an association between contact with aniline dye and the development of bladder cancer in 1895 (71). Historically bladder cancer was regarded as a neoplastic disease strongly linked to professional and environmental contact with chemicals. Two decades later, Yamagiwa and Ichikawa successfully produced a carcinoma on the inner surface of the ear of the domestic rabbit with coal-tar, proving the possibility of inducing cancer in experimental animals by chemical means (72). The induction of bladder cancer in dogs by 2-naphthylamine, reported by Hueper in 1938, established the experimental basis of bladder carcinogenesis (73). Since then, various attempts to induce tumors in rodent bladders by chemicals were unsuccessful until Armstrong and Bronser induced papillomas and carcinomas through oral administration of 2-acetylaminoflourene (AAF) in CBA strain mice in 1944 (74). However, AAF, which is a pluripotent carcinogen, induced tumors in other sites of the animal including the liver, pancreas, breast and skin as well as the bladder. The search for organo-specific chemically defined bladder carcinogens achieved great success in the 1960s and early 1970s. Among those carcinogens, three were reported as being particularly effective in causing bladder tumors under the appropriate conditions: FANFT, N-butyl-N-(4-hydroxybutyl)-nitrosamine (BBN) and MNU (75,76).

Although the induction period is relatively long, the application of these chemicals provided readily available reproducible models necessary for detailed studies of the biochemical, pathobiological and immunological mechanisms involved in the pathogenesis of bladder cancer (77). Various combinations of dose, period and frequency of administering the chemicals to the animals were attempted, and the results revealed several facts: i) Within a fixed experimental period, when the total dose of the carcinogen was increased, the grade of cellular atypia and the extent of invasion by these transformed urothelial cells increased; ii) when the total dose and frequency was fixed, the longer the inducing period, the more invasive those tumors became; iii) when the total dose and period was fixed, a greater carcinogenic effect was observed when the total dose was administered as several fractions, which means the effect of the fractions was synergistic rather than additive (76,78,79).

Of the three organo-specific carcinogens, the usage of FANFT is not currently common as a result of concern for environmental pollution and personnel health in spite of the fact that the commonly used AY-27 and MBT-2 rodent UCC cell lines, which are applied in the aforementioned syngeneic models, were induced by feeding rodents with FANFT. Thus the focus is on BBN and MNU. Since the chemically-induced tumors grow intravesically, the detection methods are the same as those employed in the transplantable syngeneic models.

### BBN

Highly limited to the urinary bladder, BBN is probably the most commonly referenced experimental carcinogen. Rodent bladder tumors induced by BBN mirror their human counterparts histologically and genetically (80). A previous study compared the mRNA and protein levels of the rodent bladder cancer model with human bladder cancer, finding concordant changes in several genes/proteins, demonstrating that the bladder cancer model induced by BBN is a powerfully reliable study tool (81).

In the mid-1990s, clinical trials demonstrated that regular non-steroidal anti-inflammatory drug (NSAID) use significantly prevented and reversed esophageal and colorectal cancer (82). Similar reports later confirmed this finding, and the chemoprevention effect covered urinary bladder cancer (83). The effect of NSAIDs, including aspirin, celecoxib, rofecoxib, naproxen and indomethacin, was evidenced by results in the BBN-induced models (84–89). Other agents, including histone deacetylase inhibitor (90), sulforaphone (91), sirolimus (92), atorvastatin (93), and natural extracts or compounds including green tea polyphenol (94), cranberry juice concentrate (95), aqueous extract of sclerotia of *Polyporus umbellatus* Fries (96) and isothiocyanates (97) have also been demonstrated to possess efficacy in the prevention of bladder tumor development, although some herbal extracts were revealed not to be as effective as previously thought (98). Studies of chemoprevention with BBN-induced models continue to contribute to understanding when transgenic mouse models are feasible.

BBN is a yellow oily liquid usually dissolved in drinking water at the concentration of 0.05% to feed the animals. Although convenient, this method exhibits problems when measuring the exact dose of BBN taken by each individual animal. Different individuals may consume different doses, and carcinogenesis may take a longer period of time in those that drink less water. Certain authors suggested gavage three times per week, solving the problem of dosing, but also requiring a far more manual operation. The incidence rate of tumor formation can be 100%, however, subsequent to feeding with BBN for 6–8 weeks, another 6–8 months are required for the development of papilloma and carcinoma in all the animals (79).

With regard to the development of squamous cell carinoma, the percentage of squamous elements varied in different reports (99,100). The variation could be a result of divergence in strains of rodents, length of inducing period and extent of chronic inflammatory reaction (101). Reproducibility is greatly hampered by the long inducing period for tumor development. Therefore, these models are more often employed in chemoprevention studies, as introduced previously, but are less practical in therapeutic efficacy studies of novel anti-proliferative compounds.

### MNU

MNU is the only carcinogen recognized to act directly on the urothelium following spontaneous pH-dependent decomposition without requiring metabolic activation. Therefore the carcinogen can be administered directly in quantifiable pulse doses via intravesical instillation, possessing unique advantages for the experimental analysis of complete carcinogenesis. MNU is a fine yellowish crystalline powder that is intrinsically unstable. As a result, variations in carcinogenic potency were observed in early experiments. Initially introduced by Hicks and Wakefield in the 1970s (102), MNU was revealed to be the fastest in carcinogenic induction. Bladder tumors appeared from 12 weeks onwards. The method was later further modified by Steinberg (103), using a different inbred strain of rat (Fischer 344) and administering continuous antibiotics in the drinking water of the animals during carcinogen exposure to minimize urinary infections, which was believed to affect the type of pathological development. The frequency he used, which was once every two weeks for four times, gradually became routine procedure (103). Originally used as a denucleator, it was later realized that MNU is also a genotoxic carcinogen, and the only carcinogen to produce bladder cancer at a single dose (104). Due to the instability, great care should be taken during its storage, preparation and use. Low temperature, protection from light and addition of 5% acetic acid are common requirements to keep MNU from decomposition.

In BBN induction, the two genders of rats or mice could be used, and male rodents are used more often, perhaps since in the human population males have a much higher incidence of bladder cancer, and notably previous findings indicate androgen or androgen receptors may be a contributing factor in tumorigenesis and angiogenesis (105,106). In MNU induction, mice are not a feasible option since the urethra is too thin for catheterization. Female rats are preferred for their anatomic structure. However, even with these, the operator would need a lot of practice prior to skillfully performing the operation, which is relatively more difficult and complicated than gavage. Urinary catheterization can occasionally cause urinary infection, urocystitis, bladder concretions and sepsis if the catheter is not inserted properly (107). Furthermore, anesthesia is also required. Some rats accidentally die due to these factors and this should be taken into consideration when designing a study.

The inherent inconvenience of the practice renders MNU less popular than BBN. However, findings are also generated using MNU-induced models, such as the chemopreventive effect of curcumin (108). Few studies have attempted other routes of administration such as intraperitoneal injections (109,110). However, in these experiments MNU was used in combination with BBN intake in order to enhance and accelerate the carcinogenic effect of the latter. The effect of intraperitoneal injection of MNU alone is questionable.

Squamous tumors were also reported in MNU-induced models. Steinberg suggested adding antibiotics in the drinking water of rats when they are administered MNU intravesical instillation to minimize the inflammatory response in order to prevent the development of squamous tumors (103). The modification resulted in 100% formation of UCC papilloma and carcinoma without squamous cell carcinoma.

## 5. Transgenic models

The transgenic mouse, or genetically engineered mouse (GEM), generated by ever-improving techniques to carry cloned oncogenes or lack tumor-suppressing genes, provides an ideal system for dissecting the roles of these molecular events, individually or in combination, in bladder tumorigenesis. In 1974, Rudolf Jaenisch created the first GEM by microinjecting a simian virus 40 DNA into an early-stage mouse blastocyst, which was subsequently transferred surgically to the uterine horns of a surrogate mother (111). During the early 1980s the technology used to generate GEMs was improved into a tractable and reproducible method (112).

A number of GEM models of UCC employed the uroplakin II (UPII) promoter, the 3.6 kb 5′-upstream sequence of the mouse *UPII* gene, first identified in 1995 (113). UPs are a group of integral membrane proteins that are synthesized as the major differentiation products of the urothelium. The UPII promoter drives a Lac Z reporter gene and a human growth hormone gene to express at a high level in the urothelium. Later it was found to be capable of driving the urothelium-specific expression of SV40T (an oncoprotein that can inactivate the p53 and retinoblastoma proteins) antigen following microinjection of the UPII-SV40T chimeric gene fragment into the pronuclei of fertilized eggs of FVB/N inbred mice for GEM production. The GEM developed UCCs that bear a strong resemblance, not only in phenotypes, but also in the mode of progression, with human UCCs (114,115). Certain GEM UCCs were even muscle-invasive. The procedure is relatively technically demanding and complex, and the target gene could only be incorporated into ~10% of produced mice.

By then, clinical and pathological studies had already identified the low- and high-grade forms of UCC, arising from two separate pathways (116). GEM UCC models were later validated and elucidated the divergent pathways. The role of numerous genes and receptors, e.g., Hras, p53, RB, PTEN, fibroblast growth factor receptor, and epidermal growth factor receptor, in the development of bladder cancer, also become more evident with the increasing insight provided by the GEM models (117–124).

However, the findings of Ayala de la Peña *et al* demonstrated that, during the original cloning of this promoter, ~1,500 bp of the UPII promoter region was oppositely inserted between two *Sac*I restriction enzyme sites (from −1262 to −2805 from exon 1) (123). Using the appropriate promoter (referred to as UPKII), expressing the SV40 large T-antigen (which inactivates both p53 and Rb) results in the development of carcinoma *in situ* only, without progression to invasive cancer. By contrast, SV40 mice created using the UPII promoter develop muscle-invasive disease (114,115). This lead to suspicion of the validation and accuracy of the results generated in GEM models with the UPII promoter, and the findings generated using the UPII promoter should be reconfirmed with the application of the appropriate promoter.

GEM models, however, have certain limitations. As a result of the inherent flaws of the mechanism, only a portion of the animals exhibit the desired genotypic traits, and occasionally it cannot be ascertained as to whether the trait is as required, as discussed above. A paucity of models representing the high-grade, muscle-invasive and metastatic form of bladder cancer remains. With target genes switched on or off, GEM is ideal for studying single or multiple gene functions, but any specific model may not fully mirror the genetic alterations in natural human tumorigenesis, which involves the deregulation of multiple signaling pathways. Cancer cells in these models, which have the same or similar origin, tend to be less heterogeneous than human bladder cancer. Therefore, the biological behavior of the cancer may be different from the human case. GEM is usually not applied in testing the efficacy of novel therapeutic or preventive agents. When translating the laboratory findings of certain therapeutic strategies using GEM models, caution is essentially required.

In previous years, studies had applied BBN or MNU to GEM to test whether certain genes could facilitate the carcinogenesis process and shorten the long inducing period. P27 knock-out mice are more sensitive to MNU, while Stat3-transgenic mice, Fez1/Lzts1-deficient, Keap1 knock-out and FHIT knock-out mice are more susceptible to BBN (89,124–127). As the inducing period was curtailed, studies using carcinogen-induced GEM models could save time, which combines the advantages of the two types of model.

## 6. Use of rat or mouse model

Rats and mice, as important tools of study, were often used in setting up bladder cancer models. In a certain type of model, one of the animals may have preference over the other rodent. For studies of carcinogen-induced bladder cancer models, rats are more frequently used than mice, particularly when induced by MNU. By contrast, in studies of genetically modified animal models, the usage of rats is rare.

The differences in pathologic structural characteristics between rats and mice have been previously identified (14). A number of exophytic tumors induced in rats are polypoid, often pedunculated and with an inverted papillary growth pattern (14). By contrast, in mice, nodular hyperplasia is considerably more common than papillary proliferations, which were absent in a previous study (128). Thus, the rat model strongly resembles papillary neoplasms and the mouse model resembles flat urothelial lesions. Although the two forms are observed in human patients, papillary neoplasms definitely have higher incidence rates.

Since nude mice were identified as a potent tool for establishing xenograft models, mice began to gain more popularity compared to rats. This was aggravated by the emergence of GEM. Mice also have the inherent advantage of smaller size and faster breeding. However, the rat remains noteworthy in areas where its larger body size and physiological similarity to humans are important, including pharmacological studies which test the effects and toxicity of drugs. The genome sequencing study of the rat in 2004 (129) revealed that as many as 90% of rat genes exhibited matches in humans and mice, higher than the 80% reported (130) when comparing mice with humans, thus helping to restore favor for the rat in the laboratory and promoting the identification of new genetically engineered strains. In previous years the genetically modified rat became technically feasible and economically attractive (131,132). Currently available knock-out rat disease models for Parkinson’s and Alzheimer’s diseases, hypertension, and diabetes are developed using zinc-finger nuclease technology. However, bladder cancer rat models have yet to be successfully established.

## 7. Conclusion

All the models developed by scientists make an effort to maximize the approximation of reality. Since it is hard to meet with all the standards of an ideal animal model, the objective of the present critical review is not to focus on the weakness of each model, dissert the superiority of one model over another, or prove the insufficient validation of the findings reported by studies using these models, but to offer investigators more information to take into account and select the ideal model that suits the relevant question of interest. Although GEM cancer models have been developing rapidly and showing great potential, they cannot fully take the position of transplantable and carcinogen-induced models. A meta-analysis compared outcomes from autochthonous models and GEMs and revealed that carcinogen-induced tumors showed an improved association with clinical responses (133).

Certain studies recruit more than one type of animal model to enhance its validity and translatability. In an investigation of the chemopreventive and chemotherapeutic effects of silibinin, the heterotopic xenograft and MNU induced models were employed (134). Perhaps multi-animal model studies will become a trend in the future.

Animal models of bladder cancer have advanced the understanding of the disease, enabled the exploration of treatment regimens and, ultimately, the translation of preclinical knowledge to improved patient care. Further effort should be devoted to modifying and optimizing these models. Every protocol must be approved by the institutional ethics committee for the use of laboratory animals to protect the welfare of the animals.
